# mCRP-Associated Vascular Pathophysiology in Progression and Outcome of Intracerebral Hemorrhage

**DOI:** 10.3390/ijms26136195

**Published:** 2025-06-27

**Authors:** Gabriela Șalari, Mark Slevin

**Affiliations:** 1 Department of Anesthesiology and Intesive Care, Emergency Country Hospital Targu Mures, 540136 Targu Mures, Romania; dr.gabriela.salari@gmail.com; 2 Centre for Advanced Medical and Pharmaceutical Research, “George Emil Palade” University of Medicine, Pharmacy, Science and Technology, 540142 Targu Mures, Romania

**Keywords:** mCRP, intracerebral hemorrhage, vascular rupture, bis-phosphocholine dimer 1,6-bis(phosphocholine)-hexane

## Abstract

Monomeric C-reactive protein (mCRP), derived from the dissociation of the native pentameric CRP (pCRP), has been implicated in the pathophysiology of various neurological conditions, particularly intracerebral hemorrhage (ICH) and neurodegenerative diseases. mCRP accumulates in the brain after hemorrhagic stroke, contributing to the formation of the metabolic penumbra and promoting inflammation. Recent studies have linked mCRP to the activation of microglia, endothelial cells, and complement pathways, which collectively intensify neuroinflammation and disrupt tissue repair mechanisms. Additionally, mCRP is associated with cognitive decline, particularly in ICH survivors, by promoting microvascular damage, neurodegeneration, and vascular instability. The presence of mCRP in distant regions of the brain, including the hypothalamus, suggests its potential role in spreading inflammation and exacerbating long-term neurological damage. This review synthesizes findings on the pathogenic role of mCRP in stroke and neurodegeneration, proposing that mCRP could serve as both a biomarker and a therapeutic target for improving outcomes in stroke patients. Emerging immunopharmacological strategies are being actively pursued to mitigate the pathogenic activity of mCRP, a potent pro-inflammatory effector implicated in a variety of immune-mediated and neuroinflammatory conditions. These approaches encompass the inhibition of native pentameric CRP dissociation into its monomeric isoform, the disruption of mCRP’s high-affinity interactions with lipid rafts and cell surface receptors involved in innate immune activation, and the enhancement of its clearance through mechanisms such as solubilization, opsonin-mediated tagging, and phagocytic engagement. Targeting these immunoregulatory pathways offers a compelling therapeutic framework for attenuating mCRP-driven inflammatory cascades in both systemic and CNS-specific pathologies.

## 1. Introduction

Stroke ranks as the second most frequent cause of mortality and long-term functional impairment globally. While it does not represent a uniform pathological condition, stroke results from a diverse array of risk determinants, disease mechanisms, and predisposing medical disorders. Among these, hypertension stands out as the most readily adjustable risk factor, despite its varying degree of influence across different stroke subtypes. ICH is a devastating event, often leading to significant mortality, morbidity, and long-term cognitive impairment. One of the key factors contributing to the pathophysiology of ICH is the presence of mCRP, a structural isoform of the native pCRP. Recent studies have provided evidence that mCRP plays a crucial role in neuroinflammation, exacerbating tissue damage and contributing to the progression of neurodegenerative processes. Unlike the native pCRP that circulates in plasma, mCRP is a tissue-associated isoform generated locally at sites of vascular injury. After a hemorrhagic stroke, pCRP entering the brain parenchyma undergoes conformational dissociation into mCRP, which then binds to endothelial cells, astrocytes and neurons around the hematoma. This locally deposited mCRP triggers a cascade of innate immune activation—for example, it activates complement and endothelial signaling pathways—leading to blood–brain barrier (BBB) disruption, leukocyte recruitment and edema formation. Experimental and histological studies confirm that high levels of mCRP in perihematomal tissue exacerbate secondary injury: animal models show that mCRP infusion enlarges edema and neuronal loss, and human autopsy data reveal abundant mCRP immunostaining around the hemorrhage core. Substantial evidence indicates that mCRP is not merely a bystander but an active driver of post-hemorrhagic neuroinflammation and tissue damage, directly amplifying the severity and spread of ICH pathology.

This review explores the molecular mechanisms through which mCRP influences brain inflammation and neuronal degeneration, highlighting its potential role in the progression of cognitive decline post-stroke, and the emerging therapeutic implications associated with targeting mCRP for stroke and neurodegenerative diseases.

## 2. Monomeric CRP as a Central Player in Stroke

### Stroke Subtypes and Their Etiologies

Stroke is broadly classified as ischemic or hemorrhagic, with most strokes being ischemic due to the thrombotic or embolic occlusion of brain arteries. Atherosclerotic plaque formation is a major substrate for cerebral thrombosis [1]. mCRP has been identified as a key inflammatory effector within both ischemic and hemorrhagic brain lesions, where it interacts with endothelial and glial cells to amplify local immune responses. Its tissue deposition correlates with the activation of potent inflammatory pathways implicated in secondary brain injury. Clinically, elevated levels of acute-phase CRP have been consistently linked to larger infarct and poorer neurological outcomes in ischemic stroke, as well as to unfavorable prognoses following intracerebral hemorrhage. Furthermore, longitudinal evidence indicates that higher circulating mCRP concentrations are associated with an increased burden of subclinical vascular pathology: individuals with levels above the population median exhibited a 4.7-fold higher risk of developing new carotid plaques over a seven-year period—suggesting a direct role of mCRP in promoting atherosclerotic progression and, by extension, stroke susceptibility. These findings support mCRP not merely as a passive biomarker but as an active contributor to cerebrovascular disease [2]. Early clinical assays could not distinguish mCRP, and many immunohistochemical studies labeled all CRP in plaques—including what was actually mCRP [3].

Roughly 85% of strokes are ischemic, predominantly linked to pathologies such as small-vessel disease (arteriolosclerosis), the emboli of cardiac origin, or the atherothrombotic occlusion of major cerebral arteries. Intracerebral hemorrhages located in deep regions (such as the basal ganglia and brainstem), the cerebellum, or lobar areas constitute over 15% of all strokes worldwide. Lobar hemorrhages are commonly attributed to cerebral amyloid angiopathy or arteriolosclerotic changes, whereas deep hemorrhages are more frequently associated with hypertensive arteriopathy affecting deep perforating vessels. A smaller proportion—approximately 20%—of intracerebral hemorrhages are linked to macrovascular anomalies including aneurysms and cavernous malformations, cerebral venous sinus thrombosis, or other rare etiologies, which are particularly significant in younger individuals under the age of 50 [4].

## 3. Structural and Functional Aspects of CRP, from Pentamer to Monomer

### 3.1. CRP—A Marker of Inflammation

A substantial body of evidence suggests that inflammation plays a pivotal role in the pathogenesis of cerebrovascular disease [5]. The liver generates CRP, one of the most prominent characteristics of the acute inflammatory response. CRP is also a systemic marker of inflammation that has been widely researched [6]. Several studies have highlighted the clinical relevance of elevated CRP levels as a biomarker of systemic inflammation. Particularly noteworthy is the fact that this marker has been the subject of a substantial amount of research over the last several decades to investigate its function in the context of intracerebral hemorrhage. At the present time, it is being considered as a risk assessment instrument and a prognostic marker. Building on the role of CRP, the transition from its pentameric to monomeric form warrants examination due to its implications for functional modulation and pathophysiological relevance [7].

### 3.2. Transformation of Pentameric CRP to Monomeric CRP

pCRP acts as the biological precursor for modified mCRP formation. At sites of tissue damage, pCRP undergoes conversion to mCRP through interactions with membrane lipids. This process begins when pCRP binds to exposed phosphocholine groups in a calcium-dependent manner [8]. The conversion process is initiated when phospholipase A_2_ (PLA_2_) catalyzes the hydrolysis of phospholipid acyl chains, thereby exposing membrane-bound phosphocholine moieties while concomitantly generating lysophosphatidylcholine (lyso-PC)—a bioactive lipid with detergent properties. Subsequently, pCRP becomes anchored to these modified membrane domains, where its proximity to hydrophobic membrane compartments provides the necessary thermodynamic drive for pentameric dissociation. Remarkably, this structural transformation exposes a previously hidden cholesterol-binding site unique to mCRP. This novel binding domain demonstrates specific affinity for cholesterol-enriched lipid rafts—highly organized membrane microdomains that serve as crucial platforms for the spatial organization and regulation of cellular signaling cascades under both normal physiological conditions and during disease pathogenesis. The characterization of mCRP’s mechanism of action is essential to understand its structural–functional correlations and biochemical activity within inflammatory pathways [9].

### 3.3. Cellular Binding and Mechanism of Action

**Figure 1:**
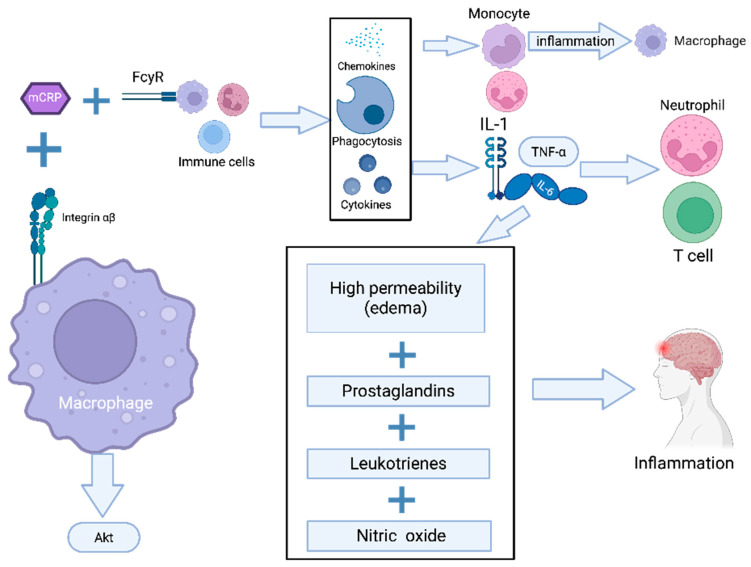
mCRP-induced activation of innate immunity and inflammatory pathways. Pathway legend: The schematic depicts the role of mCRP in activating macrophages through binding to integrin α5β3 and Fcγ receptors (FcγRs), leading to AKT pathway activation. This results in the upregulation of pro-inflammatory cytokines (IL-1, IL-6, IL-α) and chemokines, the recruitment of monocytes, neutrophils, and T cells and the enhancement of phagocytic activity. The inflammatory response is further sustained by increased vascular permeability (edema) and the release of prostaglandins, leukotrienes, and nitric oxide.

mCRP interacts with surface receptors on macrophages through α5β3 integrin binding, initiating AKT-mediated signaling cascades that establish a pro-inflammatory phenotype and enhance chemotactic responses [10]. In addition to his mechanism, CRP contributes to immune modulation through its affinity for Fcγ receptors (structurally similar to the Fc domains of immunoglobulin G), thereby stimulating phagocytic activity in macrophages and other innate immune cells [11]. A schematic representation is provided in Figure 1.

CRP serves as an effective opsonin for *Neisseria meningitidis* and various bacterial pathogens, significantly enhancing their recognition and subsequent phagocytic clearance by human macrophages and neutrophils via antibody-mediated augmentation of the engulfment process. Structural transitions in CRP, particularly the reduction in intra-subunit disulfide bridges following the calcium-dependent binding of native pCRP to cellular surfaces, expose cryptic lipid raft-targeting motifs. This conformational change dramatically potentiates cellular internalization and signal transduction in both macrophages and vascular endothelium [11]. In endothelial cells, rmCRP exhibits specific affinity for cholesterol-rich membrane microdomains, triggering cellular activation through phospholipase C-dependent pathways, *mitogen-activated protein kinase* (*MAPK*) signaling, and nuclear factor kappa-B (NF-κB) activation [11].

### 3.4. The Role of C-Reactive Protein in the Inflammatory Response

PLA_2_ or the stabilization of pCRP with bis-phosphocholine hexane (bisPC) prevented mCRP formation and significantly reduced inflammatory responses in vivo. These findings establish the pCRP-to-mCRP conversion as a critical molecular switch that localizes and amplifies inflammation at sites of tissue injury, positioning it as a novel therapeutic target. Notably, Hammond DJ and colleagues [12,13] showed that, under acidic conditions (pH~5.2–4.6), human pCRP acquires a broad new binding specificity. Using ELISA-like assays with fluid-phase CRP and six different immobilized proteins (factor H, oxidized LDL, C3b, IgG, amyloid β, and BSA), they found that CRP bound none of these targets at pH 7.0 but bound all six robustly at low pH. Importantly, biophysical assays (gel filtration, ANS fluorescence and phosphocholine binding) demonstrated that low pH did not dissociate CRP into monomers but “loosened” its pentameric structure, abolishing its phosphocholine-binding site until pH was neutralized. Furthermore, effective binding required that the target proteins themselves be exposed to acidic pH (except for factor H), implying that CRP recognizes a common “non-native” epitope produced when proteins are denatured by acid. Together, these experiments support a model in which an acid-induced conformational change in pCRP exposes a cryptic binding interface that engages a range of misfolded or denatured proteins. In other words, CRP in inflamed, acidic environments appears to act as a general sensor of damaged self—binding diverse ligands that share the acid-altered conformational motif.

Furthermore, a marked elevation in mCRP expression has been observed in the vascular structures of damaged cerebral regions. Notably, mCRP displays marked pro-angiogenic activity, distinguishing it from its native counterpart, and appears to play a role in modulating tissue viability and development through its influence on vascular remodeling and neovascularization processes [13]. Supporting these observations, Krupinski et al. [14] examined 38 human carotid endarterectomy specimens pathologically classified as ulcerated non-complicated (UNC, *n* = 19), fibrous (F, *n* = 12), or ulcerated complicated/hemorrhagic (UC, *n* = 7) plaques. Using PCR gene arrays and immunohistochemistry, they found that CRP expression was markedly increased in UNC plaques: CRP mRNA was predominantly detected in UNC versus UC (*p* = 0.001), accompanied by the highest levels of interleukins (IL-6), cyclooxygenase-2 (COX-2) and monocyte chemoattractant protein-1 (MCP-1) in these lesions. Immunostaining confirmed strong CRP in endothelial cells and inflammatory infiltrates around newly formed microvessels in UNC plaques, whereas fibrous plaques were CRP-negative, and hemorrhagic plaques showed only weak staining. From these data the authors concluded that active (ulcerated non-complicated) plaques are in an active pro-inflammatory state and that local CRP synthesis may promote neovascularization and plaque instability. Although the study did not distinguish CRP isoforms, subsequent research has established that CRP deposited in vascular lesions exists mainly as the dissociated monomeric form. mCRP is a highly bioactive mediator formed from circulating pCRP at sites of injury; it adheres within the vessel wall and drives inflammation by promoting endothelial activation, aberrant angiogenesis, leukocyte recruitment and platelet adhesion/aggregation, thereby amplifying thrombo-inflammatory processes in plaques. In summary, Krupinski et al.’s findings of local CRP upregulation in high-risk carotid plaques are consistent with a model in which locally generated mCRP amplifies the inflammatory and pro-thrombotic microenvironment of unstable atherosclerotic lesions.

mCRP may play a pathogenic role in the progression of unstable atherosclerosis and the increased risk of plaque thrombosis. Research shows mCRP significantly amplifies inflammatory activation in both lab and living systems. After intracerebral hemorrhage, it accumulates persistently in brain tissue, which may explain the sustained neuroinflammation following brain injury [15]. A notable aspect of mCRP’s pathogenic potential lies in its ability to bind selectively to components of the extracellular matrix, particularly fibronectin, a major glycoprotein found in inflamed hepatic tissue. This binding occurs through a defined peptide region and has been observed to coincide with sites of active inflammation. Functionally, this interaction enhances leukocyte attachment and stimulates the endothelial expression of adhesion molecules, thereby facilitating the recruitment of monocytes into the surrounding matrix [16]. In parallel, mCRP engages integrin receptors (such as α_vβ_3 and α_4β_1) on immune cells, activating phosphoinositide 3 kinase/protein kinase B (PI3K/AKT)-dependent chemotactic pathways [10]. Together, these mechanisms support a persistent inflammatory environment, which is believed to contribute to liver fibrogenesis by promoting sustained immune cell activation and matrix infiltration [17].

Post-ICH studies reveal CRP concentrated in blood vessels and inside activated astrocytes and neurons surrounding the hemorrhage site [18]. The neurotoxic potential of mCRP is increasingly recognized, particularly in the context of blood–brain barrier disruption, where it gains access to neural tissue and exerts direct pathological influence. Unlike its pentameric counterpart, mCRP actively alters neuronal homeostasis. Experimental data reveal that its presence amplifies key processes involved in neurodegeneration, notably the accumulation of β-amyloid and the hyperphosphorylation of tau protein—both hallmark features of Alzheimer’s (AD)-like pathology. These effects are not uniform across all genetic backgrounds but appear markedly intensified in the presence of the *apolipoprotein E*, *allele 4* (*APOE4*) allele, suggesting a synergistic vulnerability in genetically predisposed individuals. This genotype-specific response positions mCRP as a potential molecular bridge between vascular compromise and progressive neurodegenerative cascades [19].

Nevertheless, these observations do not exclude a systemic origin of CRP. Research suggests that CRP may cross into brain tissue from the circulation by inducing endothelial contractile activity, as evidenced by both in vitro BBB models and ex vivo whole-brain preparations [20].

In summary, current findings support the hypothesis that native pentameric CRP may act as an extracellular chaperone, mitigating the detrimental effects of protein misfolding and aggregation in acidic inflammatory microenvironments. Upon dissociating into mCRP in areas of inflammation, it deposits within the brain parenchyma at the extracellular matrix (ECM) level, where it exerts pro-inflammatory effects that enhance and localize the inflammatory response. A precise understanding of CRP’s conformational dynamics is essential for delineating its structure–function paradigm and its mechanistic involvement in inflammation at the molecular level [20].

## 4. The Multifaceted Role of mCRP

### 4.1. mCRP in Cardiovascular Diseases and Stroke

CRP and the serum amyloid P (SAP) component are the main pentraxins (PTXs) found in humans. As part of an evolutionarily conserved protein family, pentraxins play a key role in the immune response. Structurally, they are characterized by a pentameric arrangement and bind to their target ligands through a calcium-dependent mechanism [21]. CRP and SAP are soluble proteins that recognize pathogenic germs or injured cells. They interact with the complement cascade and Fcγ receptors to activate the innate immune system [22]. CRP, also known as PTX1, occurs in two physically and functionally separate forms: (1) net anti-inflammatory serum-associated native pCRP and (2) pro-inflammatory tissue-associated mCRP [23]. The development and characterization of antibodies that can distinguish between native CRP and mCRP have significantly advanced the ability to detect physiologically active mCRP in tissues [24].

Strang et al. [25] investigated the interaction between amyloid-beta (Aβ) plaques and CRP in AD. Their study demonstrated that Aβ plaques induce the dissociation of pCRP into its mCRP, which has distinct pro-inflammatory properties. In vitro experiments revealed that only Aβ plaques, and not non-aggregated Aβ peptides, triggered this dissociation. An immunohistochemical analysis of post-mortem frontal cortex sections from AD patients showed significantly higher levels of mCRP compared to controls, with no significant difference in pCRP levels. Furthermore, mCRP was found to co-localize with Aβ plaques and the complement component C1q, indicating potential activation of the complement system. The authors propose that Aβ plaques may displace calcium ions bound to pCRP, leading to its dissociation into mCRP, thereby localizing and amplifying inflammation in AD-affected brain regions.

Marchesi et al. [26] proposed a vascular hypothesis for AD, suggesting that the disease originates from oxidative-induced inflammation and dysregulated amyloid metabolism in small blood vessels. He emphasized that early pathological changes in AD involve oxidative stress-induced damage to the microvasculature, leading to ischemia. This ischemic environment activates amyloid-processing enzymes and pro-inflammatory factors, which, over time, compromise neuronal functions and contribute to the complex lesions characteristic of advanced AD. He also speculated that low-abundance, gain-of-function somatic mutations of the *amyloid precursor protein* (*APP*) may play a role in triggering this pathological cascade.

The study by Bulbarelli et al. [27] investigates the effects of oxygen and glucose deprivation (OGD) on amyloid-β_42_ (Aβ_42_) production in brain capillary endothelial cells. The authors demonstrate that OGD induces a significant increase in Aβ_42_ peptide production through a mechanism involving the upregulation of β-secretase (*BACE1*) via hypoxia-inducible factor-1 (HIF-1) activation. Additionally, they observe a time-dependent increase in *APP* gene and protein expression, confirming the presence of *APP* in the cerebrovascular domain. These findings suggest that ischemic events can directly enhance amyloidogenic metabolism in endothelial cells, potentially contributing to impaired Aβ clearance and blood–brain barrier dysfunction in AD.

The brain extracellular matrix of patients after acute ischemic stroke clearly overexpressed mCRP [28]. Furthermore, mCRP was found to co-localize with CD105 in microvessels, indicating a role in angiogenesis. Phospho-protein array analysis and Western blotting revealed the activation of signaling pathways in both endothelial cells and neurons, as evidenced by phosphorylated insulin receptor substrate 1 (p-IRS-1), phosphorylated Tau (p-Tau), and phosphorylated extracellular signal-regulated kinases 1/2 (p-ERK1/2). This activation was inhibited following pre-incubation with mCRP antibodies, suggesting the potential therapeutic value of the antibody. The vascular system is clearly affected abnormally, as mCRP induces hemorrhagic angiogenesis in mice with matrigel implants and alters the permeability of the vascular monolayer and gap junctions [5].

Persistent inflammation after stroke linked with persistent cerebrovascular dysfunction may produce β-amyloid deposition in damaged regions; this might be sustained at least in part by the presence of too high mCRP [29]. Likewise, the accumulation of β-amyloid is known to directly cause neuroinflammation, hence perhaps extending the neurodegenerative effects [30]. mCRP is known to induce aberrant angiogenesis so its expression may have a deleterious impact on either the current or neo-microvessel function and patency, helping to produce a local hypoperfused neurodegenerative-friendly environment [31]. The localization of mCRP with phosphorylated tau in neurons might be important physiologically. Others and we have demonstrated that mCRP directly phosphorylates tau in vitro (Ser 202, 396), likely through a mechanism involving glycogen synthase kinase 3 (GSK3). It is interesting to note that, although colocalizing the two proteins, mCRP and p-Tau, they were typically at separate locations within the neurons. This nonetheless implies that mCRP may be involved in the aberrant activation of these neurons after stroke [31]. Overall, mCRP was not detected in the normal-looking brain tissue of non-dementia patients; nonetheless, it is generated and laid down in great numbers inside the brain after stroke, other brain damage, or conditions connected with neuroinflammation. This protein clearly contributes to the development of AD and vascular dementia because it has important unusual biological characteristics related to nerve cell signaling, blood vessel control, and the formation of new blood vessels, as well as its direct involvement in inflammatory responses [13].

mCRP plays a central and distinct role in vascular inflammation, contributing to key mechanisms underlying cardiovascular and cerebrovascular pathology, including endothelial dysfunction, thrombosis, and plaque instability. Despite growing evidence of its pathogenic potential, mCRP remains a largely untapped therapeutic target. Advancing mCRP-specific diagnostics and interventions represents a promising yet underdeveloped avenue to address residual inflammatory risk and improve clinical outcomes in stroke and cardiovascular disease.

### 4.2. Epidemiological Studies of CRP and ICH Risk

Approximately 20% of cerebrovascular events are attributed to spontaneous intracerebral hemorrhage, a subtype of stroke marked by disproportionately high mortality rates and substantial long-term neurological sequelae among survivors [32]. ICH typically lacks discernible prodromal manifestations, distinguishing it from ischemic stroke and rendering its pre-event detection particularly problematic. As a result, the clinical imperative to identify vulnerable individuals remains unresolved. Epidemiological studies have correlated a higher likelihood of ICH with non-modifiable factors such as age and sex, as well as modifiable ones including hypertension, diabetes, excessive alcohol intake, and smoking [33]. Existing predictive models grounded in traditional risk parameters fail to offer sufficient precision, necessitating the exploration of alternative early intervention strategies. Low-grade systemic inflammation is increasingly understood as a unifying factor in the pathogenesis of multiple disease states, and corresponding serum markers of inflammatory burden have demonstrated significant prognostic value in cardiovascular disease progression and survival outcomes [34]. The concentration of serum CRP exhibits a well-established association with increased susceptibility to coronary heart disease, ischemic stroke, and vascular-related mortality, underscoring its role in inflammatory-mediated vascular pathology [35]. In contrast, no conclusive relationship has been established between serum CRP levels and intracerebral hemorrhage risk. Despite multiple large-scale, multiethnic epidemiological studies conducted during the last decade, a meaningful link remains unsubstantiated.

The study by Bos et al. [36] aimed to assess whether high-serum CRP levels independently predict stroke risk in the general population. Elevated serum CRP levels have been shown to be significantly associated with an increased risk of both overall and ischemic stroke. However, findings from a large prospective cohort within the Rotterdam Study, encompassing 6430 stroke-free individuals followed for an average of 8.2 years, indicate that the inclusion of CRP measurements into established stroke risk prediction models, such as the Framingham Stroke Risk Score, does not enhance predictive accuracy. These results suggest that, while CRP is correlated with stroke incidence, its clinical utility as an independent biomarker for improving individualized stroke risk assessment remains limited

Chei et al. [37] conducted a nested case–control study within the Circulatory Risk in Communities Study (CIRCS) to examine the association between hs-CRP levels and the risk of stroke and its subtypes in a Japanese population. A population-based study including 13,521 participants aged 40 to 85 years reported 261 incident strokes by the conclusion of 2005. Elevated levels of hs-CRP were significantly associated with an increased risk of total stroke, ischemic stroke, and lacunar infarction, even after adjustment for established cardiovascular risk factors. Each standard deviation increment in hs-CRP corresponded with a 17% rise in total stroke risk and increases of 27% and 24% in ischemic stroke and lacunar infarction risks, respectively. Conversely, no statistically significant correlation was detected between hs-CRP concentrations and hemorrhagic stroke risk. These findings support the role of hs-CRP as a predictive biomarker for ischemic stroke subtypes in the studied Japanese population.

There is growing recognition that CRP may serve as an independent predictor of outcomes in patients with ICH, offering a convenient and measurable parameter in prognostic models. Despite this, results remain inconclusive across different ethnic groups, suggesting that CRP may differentially reflect pathophysiological subtypes of ICH depending on genetic, sex-related, and environmental influences. This variability underscores the need for cautious interpretation when using CRP in clinical assessments. In parallel, the impact of CRP gene variants on its biological activity and whether such polymorphisms act independently or synergistically remains unclear and merits focused investigation. Before CRP can be integrated into standard prognostic frameworks, enhancements in assay technology are necessary. Current high-sensitivity platforms fail to discriminate between CRP isoforms—an omission that may limit their relevance in hemorrhagic stroke. Advancing the field will require the development of isoform-sensitive assays and further studies to evaluate their diagnostic and therapeutic value in the ICH context [3].

### 4.3. Anatomo-Pathological Study in ICH

The clinical impact of intracranial hemorrhage is profound, with significant rates of morbidity and mortality stemming from its multifactorial complications. mCRP, the pro-inflammatory isoform derived from native CRP, is released in considerable quantities during inflammatory and ischemic events. Evidence suggests that mCRP may play a role in long-term neuropathological alterations, potentially affecting structurally remote brain regions years after the primary insult [38]. In the context of ischemic stroke, mCRP has been shown to localize extensively in the parenchymal compartment—particularly within neuronal nuclei, cytoplasm, and angiogenic microvessels expressing CD105. This spatial distribution is most pronounced in zones of maximal damage, including both the infarct core and the surrounding penumbra [28].

The persistent elevation of mCRP levels has been documented for months following cerebrovascular injury. In the context of intracerebral hemorrhage, mCRP is markedly expressed in the perihematomal parenchyma and has been detected in both neurons and glial cells in early post-mortem examinations, particularly in cases with fatal outcomes occurring within 12 h of symptom onset. Moreover, systemic levels of pCRP have shown predictive value for hematoma expansion and prognosis. Histopathological evidence further indicates the robust deposition of mCRP in microvascular structures within the perihematomal metabolic penumbra in patients who died within two days post-ICH. Mechanistically, mCRP is believed to enhance vascular permeability and stimulate aberrant angiogenic pathways, contributing to destabilization of the vascular architecture and increasing susceptibility to secondary hemorrhagic transformation [18].

### 4.4. The Role of mCRP in Hemorrhagic Stroke and Hypothalamic Inflammation

In the aftermath of ICH, mCRP is deposited locally in the vascular and parenchymal compartments, where it interacts with endothelial and immune receptors, initiating a cascade of intracellular signals that perpetuate inflammation. Experimental models reveal that mCRP—distinct from its pentameric counterpart—activates the *p38 mitogen-activated protein kinase* (*p38 MAPK*) pathway in endothelial cells, resulting in the overexpression of chemokines such as *chemokine* (*C-C motif*) *ligand 2* (*CCL2*) and *IL-8*, as well as key adhesion molecules—*intercellular adhesion molecule 1* (*ICAM-1*), vascular cell *adhesion molecule 1* (*VCAM-1*), and E-selectin—thereby facilitating leukocyte recruitment and disruption of the BBB [39]. Simultaneously, *p38 MAPK* activation contributes to endothelial cell apoptosis by suppressing protective mediators like *vascular endothelial growth factor* (*VEGF*) and COX-2 while increasing *ICAM-2* and *VCAM-1* levels—effects shown to be reversible with pharmacological p38 inhibition [40].

**Figure 2:**
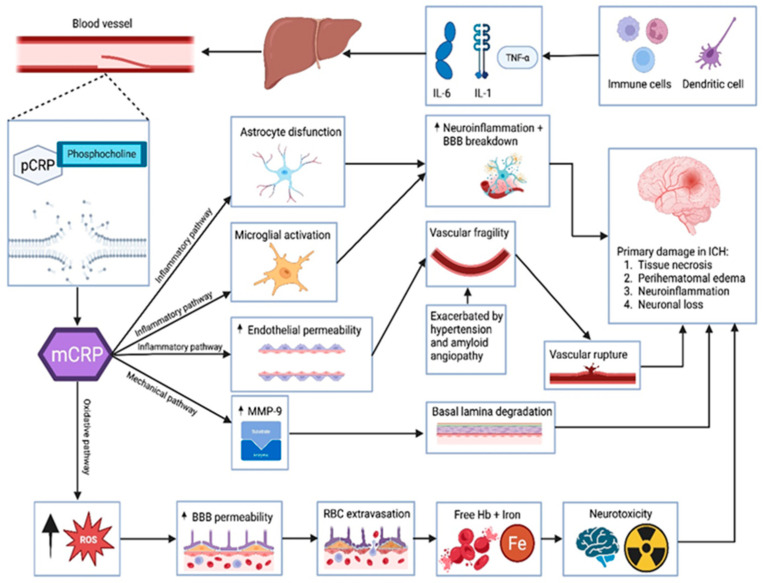
Pathogenic mechanisms linking CRP isoforms to primary brain injury in hemorrhagic stroke. Legend: Illustration of the pathogenic cascade in hemorrhagic stroke, highlighting the role of CRP isoforms. Systemic inflammation, mediated by IL-6, IL-1β, and TNF-α released from immune cells and dendritic cells, stimulates the hepatic synthesis of pentameric CRP. Upon encountering exposed phosphocholine on injured vascular membranes, pCRP dissociates into mCRP, which, via inflammatory, mechanical, and oxidative pathways, promotes locally astrocyte dysfunction, microglial activation, high endothelial permeability, MMP-9 release, and oxidative stress. These processes contribute to BBB disruption, vascular fragility, and ultimately to parenchymal damage via tissue necrosis, perihematomal edema, neuroinflammation, and neuronal loss in the setting of intracerebral hemorrhage.

This inflammatory amplification is not confined to the local hemorrhagic site. mCRP is involved in a broader pro-inflammatory loop via Fcγ-receptor and complement pathways, which exacerbate secondary damage. This is reflected in vivo through the expansion of hematoma, the intensification of edema, and heightened M1-type microglial activity marked by the elevated expression of inducible nitric oxide synthase (iNOS), NOD-like receptor family, *pyrin domain containing 3* (*NLRP3*) inflammasome components, and COX-2 [40]. Furthermore, immunohistochemical findings from animal models suggest that mCRP may travel through cerebral microvessels and reach distant targets such as the hypothalamus, potentially influencing neuroinflammatory networks beyond the primary injury site. Although the exact molecular mechanisms in these distal regions remain insufficiently defined, current evidence underscores mCRP’s central role in amplifying cerebrovascular inflammation through the *p38/MAPK* axis and downstream inflammatory mediators [39,40]. Figure 2 illustrates pathogenic mechanisms linking CRP isoforms to primary brain injury in hemorrhagic stroke

Furthermore, the detection of mCRP in anatomically distant brain structures, particularly within the hypothalamus, implicates it in long-range neuroinflammatory signaling. Given the hypothalamus’s role in regulating metabolic and autonomic functions, such inflammation may underlie systemic complications frequently observed post-stroke, including metabolic syndrome and cardiovascular dysregulation. While the pathways through which mCRP reaches these distant regions remain to be elucidated, its presence reinforces the hypothesis that mCRP is not merely a byproduct of injury but an active propagator of neurovascular dysfunction and secondary degeneration. Although elevated circulating CRP levels have been linked to poor outcomes and hematoma expansion, the specific contribution of locally expressed mCRP warrants further clarification. A major limitation in the current body of evidence is the inability of standard assays to differentiate between CRP isoforms, impeding the clinical translation of these findings. Additionally, the causal relationship between mCRP and downstream neurodegenerative or metabolic processes remains incompletely defined.

Nonetheless, the dual role of mCRP—as both a potential biomarker and a mechanistic contributor to post-ICH inflammation—offers a compelling rationale for continued investigation. Future work should prioritize the development of isoform-specific detection tools, the characterization of mCRP’s kinetics in human stroke, and the exploration of targeted anti-mCRP strategies. Such efforts may ultimately lead to novel therapeutic approaches aimed at reducing inflammation-driven damage and improving outcomes in patients with hemorrhagic stroke.

### 4.5. mCRP in Hemorrhagic Stroke: A Key Player in Neurodegeneration and Cognitive Decline

Previous research suggests that neuronal mCRP expression is significantly associated with the presence of microbleeds in the brain [41]. Specifically, patients exhibiting more than four microbleeds on computed tomography (CT)/magnetic resonance imaging (MRI), confirmed by subsequent pathology, showed higher levels of neuronal mCRP expression. This correlation is particularly noteworthy since an increasing number of microbleeds have been linked to more severe cognitive impairments [42]. Notably, survivors of intracerebral hemorrhage experience high rates of cognitive impairment. Early-onset dementia after ICH is strongly associated with hematoma size and location, while delayed dementia, which is common among ICH survivors, is less correlated with the immediate characteristics of the hemorrhagic event [43]. The delayed expression of neuronal mCRP suggests that it functions not only as a mediator of the stress response following ICH but also as a key factor in neurodegeneration, related to the chronicity and prolonged progression of microbleeds. This points to heterogeneous biological mechanisms that contribute to the delayed cognitive decline observed in ICH survivors. Regarding endothelial mCRP expression, it appears to be primarily driven by ischemia and is detectable early in ischemic stroke.

Reactive astrocytes, expressing *aquaporin 4* (*AQP4*) and mCRP, were found near large hematomas, which strongly indicates their role in facilitating water entry into the perihematomal area and contributing to brain edema, a major cause of stroke-related mortality. Additionally, since *AQP4* is part of the glymphatic system—a brain-wide network of channels responsible for clearing toxic proteins via cerebrospinal fluid—it may help clear mCRP deposition in perihematomal zones, thereby reducing tissue damage. This seems to be the first report of co-deposition of mCRP and *AQP4* in brain tissue after ICH. Furthermore, the loss of *AQP4*’s perivascular localization may make the aging brain more susceptible to the aggregation of toxic proteins like amyloid-β, contributing to neurodegenerative diseases [44]. Recent research has further established the role of mCRP in neuroinflammation, particularly in the damaged brain tissue post-stroke, where it perpetuates inflammation and activates pathways linked to neurodegeneration [45]. The transformation of circulating pCRP into its pro-inflammatory isoform, followed by dissociation into mCRP, occurs on necrotic, apoptotic, and ischemic cells, β-amyloid structures, and activated cell membranes (such as those of platelets, monocytes, and endothelial cells), often involving binding to phosphocholine. This activation leads to the stimulation of platelets, leukocytes, endothelial cells, and complements [46]. mCRP is now recognized as a crucial pathogenic factor, potentially destabilizing atherosclerosis and predisposing individuals to myocardial infarction [47].

Given this evidence, inhibiting the dissociation of pCRP presents a promising new anti-inflammatory therapeutic approach. The idea that the hypothalamus functions as a sensor linking peripheral inflammation to the brain is supported by the work of Fang and Yujie [41], which reported three male patients with acute cortical infarction in the right parietal lobe, each presenting with left upper limb weakness. Despite initial normal electrocardiograms and myocardial enzyme levels, all three developed significant cardiac events: one succumbed to sudden death due to ventricular fibrillation, while the other two experienced acute myocardial infarction approximately two weeks post-stroke. The authors hypothesized that lesions in the right parietal cortex, particularly in Brodmann area 7, may disrupt autonomic regulation by overstimulating the posterior lateral hypothalamic nucleus, leading to sympathetic dominance and subsequent cardiac arrhythmias. These findings underscore the necessity for vigilant cardiac monitoring, including electrocardiography and troponin assessment, in patients with right parietal cortical infarction and contralateral limb weakness to facilitate the early detection and intervention of neurogenic heart disease. Peripheral CRP production in the liver serves as an important sensor of systemic inflammation, relaying inflammatory signals via the autonomic nervous system to the brain, making it a crucial target of the inflammatory reflex [48].

## 5. Therapeutic Strategies

Comparable findings of mCRP presence in both microvessels and larger vessels during the chronic phase of hemorrhagic stroke—primarily within the perihematomal region—have been reported, where it was associated with *AQP-4* overexpression, a marker linked to edema. Researchers have identified a potential ability of mCRP to migrate to distant brain regions, such as the hypothalamus—a finding that was experimentally reproduced in mice following stereotactic injection of mCRP into the hippocampus. This spatial dissemination is most plausibly explained by transport through the microcirculatory system, possibly facilitated by microvesicles or immune cells, allowing mCRP to exert its effects beyond the initial site of action. These observations highlight several therapeutic avenues worth exploring. Strategies may include preventing the dissociation of pCRP into mCRP within the body, blocking the interaction of mCRP with cell surfaces, or promoting the removal of mCRP aggregates via mechanisms such as solubilization, opsonization, or phagocytosis [49]. The clinical relevance and potential research directions regarding mCRP in stroke are outlined in Table 1.

The therapeutic implications of mCRP’s role in neuroinflammation following ICH remain incompletely understood, highlighting the need for targeted mechanistic exploration. One potential strategy involves disrupting mCRP transport into the brain, which could prevent its interaction with cerebrovascular and glial targets. However, the brain’s immunological compartmentalization—particularly the integrity and selectivity of the BBB—poses a significant challenge to the efficacy of peripheral CRP-lowering interventions. This emphasizes the necessity of tailoring central nervous system (CNS)-specific therapies that account for local immune signaling. Moving forward, meaningful progress will require a coordinated approach that integrates mechanistic investigations of mCRP’s inflammatory functions with translational research in preclinical models and, ultimately, clinical trials. Only through such integrated efforts can the therapeutic viability of mCRP inhibition in improving ICH outcomes be reliably determined [50].

The only small molecule currently demonstrated to inhibit the dissociation of pCRP into mCRP is the bis-phosphocholine dimer 1,6-bis(phosphocholine)-hexane (bis(PC)-H) [51]. The development of this compound was based on a strategy similar to that employed in the design of therapeutic agents targeting SAP. This method relies on crosslinking two SAP molecules using chemical moieties structurally analogous to phosphocholine head groups, which bind to the same active site. By doing so, the compound effectively interferes with lysophosphatidylcholine-mediated activation of CRP. In this context, bis(PC)-H represents a promising lead compound for future pharmacological efforts aimed at stabilizing pCRP and preventing its pro-inflammatory transformation into mCRP. Coupled with the earlier outlined therapeutic strategies—such as blocking mCRP signaling or promoting clearance of tissue deposits—such inhibitors could form the foundation of a new class of treatments targeting mCRP-mediated pathology in neurodegenerative, cerebrovascular, and cardiovascular diseases [52].

Several studies have investigated the potential role of mCRP in the treatment of patients with ICH, highlighting both its pathological significance and therapeutic potential.

Zeller, J. et al. [53] found that the small-molecule compound 10 monomeric (C10M) was specifically developed to target the PC binding site of native pCRP, aiming to prevent its dissociation into the pro-inflammatory isoforms pCRP and mCRP. While preclinical studies have reported broad anti-inflammatory effects both in vitro and in vivo, these findings remain preliminary. The translational relevance of C10M, including its pharmacokinetics, specificity, and long-term safety profile, warrants further rigorous investigation before any therapeutic application can be substantiated.

McFadyen et al. [54] identified a potential upstream approach to modulating CRP-related inflammation by using a synthetic ligand, bisPC, to maintain CRP in its native pentameric state. In the context of myocardial injury, this intervention resulted in less tissue necrosis, indicating that preventing the structural transition to monomeric CRP may blunt downstream inflammatory cascades. Translating this principle to cerebral hemorrhage, where mCRP plays a pathogenic role, the pharmacologic stabilization of pCRP could offer a strategy to restrict local inflammatory amplification. By impeding mCRP formation, agents like bisPC might suppress leukocyte trafficking and dampen complement-mediated injury in perilesional brain tissue. Although not yet validated in stroke models, this mechanism introduces a promising therapeutic direction—targeting the structural biology of CRP rather than its systemic levels.

Fujita et al. [49] reported that conformation-specific antigens offer promising targets for monoclonal antibody therapies. This study identifies mCRP as a selective target in murine arthritis and nephritis models. From screening over 1800 antibody clones, clone 3C uniquely bound the mCRP. Treatment with this antibody reduced leukocyte infiltration in an inflammation model, highlighting its therapeutic potential. Nonetheless, further research is needed to establish its safety and effectiveness in vivo. The authors suggest analogous antibodies could be used to mop up mCRP after ICH, reducing secondary injury.

At present, the most accessible and clinically validated approach to modulate CRP levels in patients appears to be the selective extracorporeal removal of CRP through targeted apheresis techniques. This method has gained attention due to its favorable safety profile and demonstrated efficacy in reducing CRP concentrations. Notably, recent clinical experiences have reported encouraging therapeutic outcomes in conditions characterized by excessive inflammation, such as acute myocardial infarction and severe COVID-19 pneumonia. These findings highlight extracorporeal CRP apheresis as a promising adjunctive treatment modality, potentially mitigating inflammatory damage by directly lowering circulating CRP. While not yet tested in ICH, this method could be applied to stroke patients: lowering systemic CRP might prevent its conversion to mCRP at the injury site, blunting systemic and neuroinflammation [55].

Intracerebral hemorrhage triggers a classic acute-phase response: serum pCRP begins to rise within hours and typically peaks in about 2–3 days. In one large human series, plasma CRP was negligible on admission but increased markedly over the first 48–72 h after ICH onset. Immunohistochemical studies show that CRP protein is detectable around the hematoma even in very early samples (e.g., within 6–12 h), but strictly mCRP—the pro-inflammatory isoform—only appears after the very acute phase [18].

Although systemic CRP rises early after intracerebral hemorrhage as part of the body’s acute-phase defense, mCRP follows a distinct timeline, appearing only later in brain regions surrounding the hematoma. This delayed local accumulation implies that mCRP is not involved in immediate pathophysiological decisions, such as surgical timing, and holds limited relevance for acute-phase diagnostics. Instead, its gradual increase aligns more closely with secondary inflammatory processes and tissue remodeling. Therefore, its clinical relevance may lie in tracking the evolution of neuroinflammation over time. Persistently elevated mCRP in the days following hemorrhage could potentially reflect ongoing injury and poorer prognosis, suggesting its use as a marker of subacute progression rather than an early intervention tool [50]. By contrast, the specific measurement of mCRP in patients has not entered practice. No published human study has validated mCRP assays for perioperative or post-surgical monitoring in ICH.

mCRP, with its pro-inflammatory activity within perihematomal brain tissue, represents a promising biomarker and therapeutic target that could refine clinical management. From a clinical perspective, the advent of robust, standardized assays capable of quantifying mCRP in plasma or cerebrospinal fluid would enable its integration into routine perioperative and postoperative monitoring. Such integration would afford several advantages:

Enhanced prognostication: mCRP quantification could improve risk stratification by identifying patients at elevated risk for hematoma expansion, perihematomal edema, and secondary neuronal injury beyond what is discernible from systemic CRP levels or conventional imaging alone.

Precision therapeutics: Real-time mCRP monitoring would facilitate the timely initiation and titration of targeted anti-inflammatory interventions, including emerging mCRP-specific monoclonal antibodies or small molecule inhibitors, thereby optimizing therapeutic efficacy while minimizing systemic immunosuppression.

Dynamic treatment monitoring: The serial assessment of mCRP levels could serve as a biomarker for therapeutic response, enabling personalized adjustments to treatment regimens and providing mechanistic insights into disease progression and resolution.

Informed surgical decision: The incorporation of mCRP status may refine indications and timing for surgical evacuation or adjunctive therapies by elucidating the inflammatory burden contributing to secondary brain injury.

The growing body of evidence places mCRP at the center of the inflammatory cascade following ICH, with clear implications for vascular instability, secondary tissue injury, and long-range neurodegenerative changes. Unlike its pentameric precursor, mCRP exhibits potent pro-inflammatory activity, particularly within perihematomal regions and distant brain structures such as the hypothalamus. Recent advances in experimental therapeutics—ranging from CRP dissociation inhibitors and neutralizing antibodies extracorporeal apheresis—underscore the translational potential of targeting mCRP as a disease-modifying approach. Despite these promising developments, therapeutic strategies remain largely preclinical, and a critical translational gap persists. Future efforts must focus on the refinement of isoform-specific detection assays, the identification of optimal therapeutic windows, and the validation of safety and efficacy in well-designed clinical studies. If successfully translated, mCRP-targeted therapies could represent a paradigm shift in the management of ICH, offering not only improved prognostication but also novel avenues for intervention against inflammation-driven neurological deterioration.

## 6. Conclusions

The evidence gathered in this review underscores the critical role of mCRP in the inflammatory and neurodegenerative processes following intracerebral hemorrhage. mCRP is not only a key mediator of local inflammation but also contributes to widespread brain damage by promoting microvascular instability, neuroinflammation, and neuronal degeneration. Its expression in distant brain regions, such as the hypothalamus, highlights its potential to propagate inflammatory cascades that contribute to long-term cognitive decline. The association between mCRP and microbleeds, as well as its contribution to neurodegeneration, positions it as a promising target for therapeutic intervention in ICH and neurodegenerative diseases. Further research is necessary to refine diagnostic tools that measure mCRP levels and assess its role in stroke. The inhibition of mCRP dissociation or blockade of its interaction with cellular targets may offer novel strategies for mitigating the secondary damage associated with ICH and preventing the progression of neurodegenerative conditions. Strategies focused on inhibiting this dissociation, blocking mCRP’s pro-inflammatory interactions at the cellular level, or facilitating its clearance from tissues represent promising avenues for drug development.

The identification of bis(PC)-H as a small molecule capable of preventing CRP dissociation further highlights the feasibility of targeting this pathway pharmacologically. Moving forward, the development of sensitive assays for accurate plasma quantification of mCRP, alongside the exploration of CRP-binding modulators, will be essential. These efforts may ultimately lead to the establishment of novel, targeted therapies with the potential to significantly alter disease progression and improve clinical outcomes in mCRP-associated pathologies. One principal avenue under investigation involves inhibiting the structural dissociation of nCRP into its monomeric isoform, thereby curbing the generation of the pro-inflammatory species at sites of vascular injury. Molecules designed to stabilize the native pentameric configuration or to selectively obstruct the conformational transition may serve as upstream modulators of mCRP bioactivity.

Alternatively, therapeutic strategies may be considered to impede mCRP’s interaction with cellular targets, particularly by blocking its insertion into lipid rafts or interfering with its binding to immunoreceptors such as FcγRIII (CD16). By interfering with these interactions, it may be possible to suppress the mCRP-driven activation of microglia, endothelial cells, and infiltrating leukocytes—key contributors to the neuroinflammatory milieu following stroke. A third therapeutic axis focuses on facilitating the clearance of mCRP aggregates from affected neural tissue. This may be achieved via immunologically mediated mechanisms, including opsonization, phagocytosis, or enzymatic solubilization, aimed at reducing the sustained pro-inflammatory burden imposed by mCRP deposition.

Future research should focus on developing validated mCRP assays for clinical use, elucidating the timing and mechanisms of mCRP-mediated damage, and conducting rigorous clinical trials to evaluate the safety and efficacy of mCRP-directed therapies. The discovery and implementation of such targeted treatments hold significant promise for improving outcomes in ICH patients, addressing a critical unmet need in stroke management.

## Figures and Tables

**Table 1:** Proposed clinical and research implications of mCRP in stroke.

Domain	Implication
Prognosis	mCRP levels may predict lesion progression, microbleeds, and cognitive decline
Diagnosis	Co-localization with inflammatory markers could serve as a tissue-level biomarker
Therapeutics	Blocking pCRP to mCRP conversion could reduce neuroinflammation and protect brain tissue
Imaging and biomarkers	Detection of mCRP deposition might assist in identifying penumbra and high-risk brain regions
Cognitive impact	mCRP may contribute to early- and late-onset post-ICH dementia
Stroke heterogeneity	mCRP involvement may vary by ethnicity, sex, genetic background
Glymphatic clearance research	Potential new area for exploring clearance of mCRP and neurotoxic proteins

Table summarizes hypothetical implications based on current evidence and proposed mechanisms involving mCRP in stroke.

## Data Availability

No new data were created, or analyzed in this study. Data sharing is not applicable to this article.

## Abbreviations

The following abbreviations are used in this manuscript:

AD	Alzheimer’s disease
ALS	Amyotrophic lateral sclerosis
APOE4	Apolipoprotein E epsilon 4 allele
APP	Amyloid precursor protein
AQP4	Aquaporin 4
Aβ	Amyloid-beta
Aβ_42_	Amyloid-β_42_
BACE-1	Beta-site APP cleaving Enzyme 1
BBB	Blood–brain barrier
bis(PC)-H	Bis-phosphocholine dimer 1,6-bis(phosphocholine)-hexane
bisPC	Bis-phosphocholine hexane
BSA	Bovine serum albumin
C10M	Compound 10 monomeric
CCL2	Chemokine (C-C motif) ligand 2
CIRSC	Circulatory risk in communities study
CNS	Central nervous system
COX-2	Cyclooxygenase-2
CRP	C-reactive protein
CT	Computed tomography
ECM	Extracellular matrix
FcγRIII	Fc gamma receptor III
GSK3	Glycogen synthase kinase 3
HIF	1 hypoxia-inducible factor-1
Hs-CRP	High-sensitivity C-reactive protein
ICAM-1	Intercellular adhesion molecule 1
ICH	Intracerebral hemorrhage
IL-8	Interleukin 8
iNOS	Inducible nitric oxide synthase
lyso-PC	Lysophosphatidylcholine
MAPK	Mitogen-activated protein kinase
mCRP	Monomeric C-reactive protein
MRI	Magnetic resonance imaging
NF-κB	Nuclear factor kappa-B
NLRP3	NOD-like receptor family, pyrin domain containing 3 (inflammasome)
OGD	Oxygen and glucose deprivation
p38 MAPK	p38 Mitogen-activated protein kinase
PC	Phosphocholine
pCRP	Pentameric C-reactive protein
p-ERKI1/2	Phosphorylated extracellular signal-regulated kinases ½
PI3K/AKT	Phosphoinositide 3-kinase/protein kinase B pathway
p-IRS-1	Phosphorylated insulin receptor substrate 1
PLA_2_	Phospholipase A_2_
p-Tau	Phosphorylated tau
PTXs	Pentraxins
SAP	Serum amyloid P
VCAM-1	Vascular cell adhesion molecule 1
VEGF	Vascular endothelial growth factor

## References

[1] Lui F., Hui C., Khan Suheb M.Z., Patti L. (2025). Ischemic Stroke.

[2] Melnikov I., Kozlov S., Pogorelova O., Tripoten M., Khamchieva L., Saburova O., Avtaeva Y., Zvereva M., Matroze E., Kuznetsova T. (2022). The monomeric C-reactive protein level is associated with the increase in carotid plaque number in patients with subclinical carotid atherosclerosis. Front. Cardiovasc. Med..

[3] Di Napoli M., Slevin M., Popa-Wagner A., Singh P., Lattanzi S., Divani A.A. (2018). Monomeric C-Reactive Protein and Cerebral Hemorrhage: From Bench to Bedside. Front. Immunol..

[4] Murphy S.J., Werring D.J. (2020). Stroke: Causes and Clinical Features. Medicine.

[5] Aronowski J., Hall C.E. (2005). New Horizons for Primary Intracerebral Hemorrhage Treatment: Experience From Preclinical Studies. Neurol. Res..

[6] Di Napoli M., Elkind M.S., Godoy D.A., Singh P., Papa F., Popa-Wagner A. (2011). Role of C-Reactive Protein in Cerebrovascular Disease: A Critical Review. Expert Rev. Cardiovasc. Ther..

[7] Di Napoli M., Parry-Jones A.R., Smith C.J., Hopkins S.J., Slevin M., Masotti L., Campi V., Singh P., Papa F., Popa-Wagner A. (2014). C-Reactive Protein Predicts Hematoma Growth in Intracerebral Hemorrhage. Stroke.

[8] Alnaas A.A., Moon C.L., Alton M., Reed S.M., Knowles M.K. (2017). Conformational Changes in C-Reactive Protein Affect Binding to Curved Membranes in a Lipid Bilayer Model of the Apoptotic Cell Surface. J. Phys. Chem. B.

[9] Rajab I.M., Hart P.C., Potempa L.A. (2020). How C-Reactive Protein Structural Isoforms with Distinctive Bioactivities Affect Disease Progression. Front. Immunol..

[10] Fujita M., Takada Y.K., Izumiya Y., Takada Y. (2014). The Binding of Monomeric C-Reactive Protein (mCRP) to Integrins αvβ3 and α4β1 Is Related to Its Pro-inflammatory Action. PLoS ONE.

[11] Ji S.-R., Ma L., Bai C.-J., Shi J.-M., Li H.-Y., Potempa L.A., Filep J.G., Zhao J., Wu Y. (2009). Monomeric C-Reactive Protein Activates Endothelial Cells via Interaction with Lipid Raft Microdomains. FASEB J..

[12] Hammond D.J., Singh S.K., Thompson J.A., Beeler B.W., Rusiñol A.E., Pangburn M.K., Potempa L.A., Agrawal A. (2010). Identification of Acidic pH-Dependent Ligands of Pentameric C-Reactive Protein. J. Biol. Chem..

[13] Slevin M., Krupinski J. (2009). A Role for Monomeric C-Reactive Protein in Regulation of Angiogenesis, Endothelial Cell Inflammation and Thrombus Formation in Cardiovascular/Cerebrovascular Disease?. Histol. Histopathol..

[14] Krupinski J., Turu M.M., Martinez-Gonzalez J., Carvajal A., Juan-Babot J.O., Iborra E., Slevin M., Rubio F., Badimon L. (2006). Endogenous Expression of C-Reactive Protein Is Increased in Active (Ulcerated Noncomplicated) Human Carotid Artery Plaques. Stroke.

[15] Das T., Mandal C. (2004). Variations in Binding Characteristics of Glycosylated Human C-Reactive Proteins in Different Pathological Conditions. Glycoconj. J..

[16] Ullah N., Ma F.-R., Han J., Liu X.-L., Fu Y., Liu Y.-T., Liang Y.-L., Ouyang H., Li H.-Y. (2020). Monomeric C-reactive protein regulates fibronectin mediated monocyte adhesion. Mol. Immunol..

[17] Gao N., Yuan P., Tang Z.-M., Lei J.-G., Yang Z.-R., Ahmed M., Yao Z.-Y., Liang D., Wu Y., Li H.-Y. (2024). Monomeric C-reactive protein is associated with severity and prognosis of decompensated hepatitis B cirrhosis. Front. Immunol..

[18] Gao N., Yuan P., Tang Z.-M., Lei J.-G., Yang Z.-R., Ahmed M., Yao Z.-Y., Liang D., Wu Y., Li H.-Y. (2012). C-Reactive Protein in Intracerebral Hemorrhage: Time Course, Tissue Localization, and Prognosis. Neurology.

[19] Gan Q., Wong A., Zhang Z., Na H., Tian H., Tao Q., Rajab I.M., Potempa L.A., Qiu W.Q. (2022). Monomeric C-reactive protein induces the cellular pathology of Alzheimer’s disease. Alzheimer’s Dementia Transl. Res. Clin. Interv..

[20] Kuhlmann C.R., Librizzi L., Closhen D., Pflanzner T., Lessmann V., Pietrzik C.U., de Curtis M., Luhmann H.J. (2009). Mechanisms of C-Reactive Protein-Induced Blood-Brain Barrier Disruption. Stroke.

[21] Agrawal A., Singh P.P., Bottazzi B., Garlanda C., Mantovani A. (2009). Pattern Recognition by Pentraxins. Adv. Exp. Med. Biol..

[22] Lu J., Marnell L.L., Marjon K.D., Mold C., Du Clos T.W., Sun P.D. (2008). Structural Recognition and Functional Activation of FcγR by Innate Pentraxins. Nature.

[23] Wu Y., Potempa L.A., El Kebir D., Filep J.G. (2015). C-Reactive Protein and Inflammation: Conformational Changes Affect Function. Biol. Chem..

[24] Schwedler S.B., Amann K., Wernicke K., Krebs A., Nauck M., Wanner C., Potempa L.A., Galle J. (2005). Native C-Reactive Protein Increases Whereas Modified C-Reactive Protein Reduces Atherosclerosis in Apolipoprotein E-Knockout Mice. Circulation.

[25] Strang F., Scheichl A., Chen Y.C., Wang X., Htun N.M., Bassler N., Eisenhardt S.U., Habersberger J., Peter K. (2012). Amyloid Plaques Dissociate Pentameric to Monomeric C-Reactive Protein: A Novel Pathomechanism Driving Cortical Inflammation in Alzheimer’s Disease?. Brain Pathol..

[26] Marchesi V.T. (2012). Alzheimer’s Dementia Begins as a Disease of Small Blood Vessels, Damaged by Oxidative-Induced Inflammation and Dysregulated Amyloid Metabolism: Implications for Early Detection and Therapeutic Targeting. Neurochem. Int..

[27] Bulbarelli A., Lonati E., Brambilla A., Orlando A., Cazzaniga E., Piazza F., Ferrarese C., Masserini M., Sancini G. (2012). Aβ42 Production in Brain Capillary Endothelial Cells after Oxygen and Glucose Deprivation. Mol. Cell Neurosci..

[28] Slevin M., Matou-Nasri S., Turu M., Luque A., Rovira N., Badimon L., Boluda S., Potempa L., Sanfeliu C., de Vera N. (2010). Modified C-Reactive Protein Is Expressed by Stroke Neovessels and Is a Potent Activator of Angiogenesis in Vitro. Brain Pathol..

[29] Thiele J.R., Habersberger J., Braig D., Schmidt Y., Goerendt K., Maurer V., Bannasch H., Scheichl A., Woollard K.J., von Dobschutz E. (2014). Dissociation of Pentameric to Monomeric C-Reactive Protein Localizes and Aggravates Inflammation: In Vivo Proof of a Powerful Proinflammatory Mechanism and a New Anti-Inflammatory Strategy. Circulation.

[30] Hyman B.T., Phelps C.H., Beach T.G., Bigo E.H., Cairns N.J., Carrillo M.C., Dickson D.W., Duyckaerts C., Frosch M.P., Masliah E. (2012). National Institute on Aging-Alzheimer’s Association Guidelines for the Neuropathologic Assessment of Alzheimer’s Disease. Alzheimer’s Dement..

[31] Slevin M., Matou S., Zeinolabediny Y., Corpas R., Weston R., Liu D., Boras E., Di Napoli M., Petcu E., Sarroca S. (2015). Monomeric C-Reactive Protein—A Key Molecule Driving Development of Alzheimer’s Disease Associated with Brain Ischaemia?. Sci. Rep..

[32] Feigin V.L., Lawes C.M., Bennett D.A., Anderson C.S. (2003). Stroke Epidemiology: A Review of Population-Based Studies of Incidence, Prevalence, and Case-Fatality in the Late 20th Century. Lancet Neurol..

[33] Ariesen M.J., Claus S.P., Rinkel G.J., Algra A. (2003). Risk Factors for Intracerebral Hemorrhage in the General Population: A Systematic Review. Stroke.

[34] Guarner V., Rubio-Ruiz M.E. (2015). Low-Grade Systemic Inflammation Connects Aging, Metabolic Syndrome, and Cardiovascular Disease. Interdiscip. Top. Gerontol..

[35] Kaptoge S., Di Angelantonio E., Lowe G., Pepys M.B., Thompson S.G., Collins R., Danesh J. (2010). C-Reactive Protein Concentration and Risk of Coronary Heart Disease, Stroke, and Mortality: An Individual Participant Meta-Analysis. Lancet.

[36] Bos M.J., Schipper C.M., Koudstaal P.J., Witteman J.C., Hofman A., Breteler M.M. (2006). High Serum C-Reactive Protein Level Is Not an Independent Predictor for Stroke: The Rotterdam Study. Circulation.

[37] Chei C.-L., Yamagishi K., Kitamura A., Kiyama M., Imano H., Ohira T., Cui R., Tanigawa T., Sankai T., Ishikawa Y. (2011). C-Reactive Protein Levels and Risk of Stroke and Its Subtype in Japanese: The Circulatory Risk in Communities Study (CIRCS). Atherosclerosis.

[38] Al-Baradie R.S., Abdel-Hadi A.M., Ahmad F., Alsagaby S.A., Slevin M., Alturaiki W., Madkhali Y., Aljarallah B.M., Alqahtani M., Miraj M. (2022). Association of Monomeric C-Reactive Protein (m-CRP) with Hypothalamic Neurons After CRP Hippocampal Administration in a Model of Dementia. Eur. Rev. Med. Pharmacol. Sci..

[39] Khreiss T., József L., Potempa L.A., Filep J.G. (2004). Conformational rearrangement in C-reactive protein is required for proinflammatory actions on human endothelial cells. Circulation.

[40] Zhang Y., Cao H. (2020). Monomeric C-reactive protein affects cell injury and apoptosis through activation of p38 mitogen-activated protein kinase in human coronary artery endothelial cells. J. Basic Med Sci..

[41] Fang L., Yujie J. (2012). Cortical Infarction of the Right Parietal Lobe and Neurogenic Heart Disease: A Report of Three Cases. Neural. Regen. Res..

[42] Martini S., Testai F.D., Woo D., Elkind M.S.V. (2017). Systemic Inflammatory Response Syndrome, Infection, and Outcome in Intracerebral Hemorrhage. Neurol. Neuroimmunol. Neuroinflamm..

[43] Biffi A., Bailey D., Anderson C.D., Gurol E.M., Greenberg S.M., Rosand J., Viswanathan A. (2016). Risk Factors Associated with Early vs. Delayed Dementia after Intracerebral Hemorrhage. JAMA Neurol..

[44] Rasmussen M.K., Mestre H., Nedergaard M. (2018). The Glymphatic Pathway in Neurological Disorders. Lancet Neurol..

[45] Slevin M., Liu D., Ferris G., Al-Hsinawi M., Al-Baradie R., Krupinski J. (2017). Expression of Monomeric C-Reactive Protein in Infarcted Brain Tissue from Patients with Alzheimer’s Disease. Turk. J. Pathol..

[46] McFadyen J.D., Zeller J., Potempa L.A., Pietersz G.A., Eisenhardt S.U., Peter K. (2020). C-Reactive Protein and Its Structural Isoforms: An Evolutionary Conserved Marker and Central Player in Inflammatory Diseases and Beyond. Subcell. Biochem..

[47] Wang J., Tang B., Liu X., Wu X., Wang H., Xu D., Guo Y. (2015). Increased Monomeric CRP Levels in Acute Myocardial Infarction: A Possible New and Specific Biomarker for Diagnosis and Severity Assessment of Disease. Atherosclerosis.

[48] Dunn A. (2008). The HPA Axis and the Immune System: A Perspective. NeuroImmun. Biol..

[49] Fujita C., Sakurai Y., Yasuda Y., Takada Y., Huang C.-L., Fujita M. (2021). Anti-Monomeric C-Reactive Protein Antibody Ameliorates Arthritis and Nephritis in Mice. J. Immunol..

[50] Kayser S., Brunner P., Althaus K., Dorst J., Sheriff A. (2020). Selective apheresis of C-reactive protein for treatment of indications with elevated CRP concentrations. J. Clin. Med..

[51] Pepys M.B., Hirschfield G.M., Tennent G.A., Gallimore J.R., Kahan M.C., Bellotti V., Hawkins P.N., Myers R.M., Smith M.D., Polara A. (2006). Targeting C-Reactive Protein for the Treatment of Cardiovascular Disease. Nature.

[52] Caprio V., Badimon L., Di Napoli M., Fang W.H., Ferris G.R., Guo B., Iemma R.S., Liu D., Zeinolabediny Y., Slevin M. (2018). pCRP-mCRP Dissociation Mechanisms as Potential Targets for the Development of Small-Molecule Anti-Inflammatory Chemotherapeutics. Front. Immunol..

[53] Zeller J., Cheung Tung Shing K.S., Nero T.L., McFadyen J.D., Krippner G., Bogner B., Kreuzaler S., Kiefer J., Horner V.K., Braig D. (2023). A novel phosphocholine-mimetic inhibits a pro-inflammatory conformational change in C-reactive protein. EMBO Mol. Med..

[54] McFadyen J.D., Kiefer J., Braig D., Loseff-Silver J., Potempa L.A., Eisenhardt S.U., Peter K. (2018). Dissociation of C-Reactive Protein Localizes and Amplifies Inflammation: Evidence for a Direct Biological Role of C-Reactive Protein and Its Conformational Changes. Front. Immunol..

[55] Torzewski J., Brunner P., Ries W., Garlichs C.D., Kayser S., Heigl F., Sheriff A. (2022). Targeting C-Reactive Protein by Selective Apheresis in Humans: Pros and Cons. J. Clin. Med..

